# Author Correction: CD8^+^ T cell activation by murine erythroblasts infected with malaria parasites

**DOI:** 10.1038/s41598-020-77423-1

**Published:** 2020-11-13

**Authors:** Takashi Imai, Hidekazu Ishida, Kazutomo Suzue, Makoto Hirai, Tomoyo Taniguchi, Hiroko Okada, Tomohisa Suzuki, Chikako Shimokawa, Hajime Hisaeda

**Affiliations:** 1 Department of Parasitology, Graduate School of Medicine, Gunma University, Gunma, 371-8511 Japan; 2 Department of Microbiology and Immunology, Graduate School of Medical Sciences, Kyushu University, Fukuoka, 812-8582 Japan; 3 Center for Medical Education, Faculty of Medicine, Gunma University, Gunma, 371-8511 Japan; 4 Department of Parasitology, Institute of Tropical Medicine, Nagasaki University, Nagasaki, 852-8523 Japan

Correction to: *Scientific Reports* 10.1038/srep01572, published online 28 March 2013

In this Article, the legend of Figure 7e is incorrect:

“(e) Purified CD11c^+^ cells (2 × 10^4^) from C57BL/6 mice infected with the indicated parasite were pulsed with or without OVA peptide and fixed, then were co-cultured with 10^5^ OT-I CD8^+^ T cells.”

should read:

“(e) Purified parasitized (GFP^+^) or unparasitized (GFP^-^) erythroblasts (2 × 10^4^) from C57BL/6 mice infected with the indicated parasite were fixed and were co-cultured with 10^5^ Rag2^−/−^ OT-I CD8^+^ T cells.”

